# Studies on Formulation and *In Vitro* Evaluation of Floating Matrix Tablets of Domperidone

**DOI:** 10.4103/0250-474X.51944

**Published:** 2009

**Authors:** S. T. Prajapati, L. D. Patel, D. M. Patel

**Affiliations:** Department of Pharmaceutics and Pharmaceutical Technology, Shri Sarvajanik Pharmacy College, Mehsana-384 001, India; 1 Department of Pharmaceutics and Pharmaceutical Technology, Dean, Faculty of Pharmacy, Dharmsinh Desai University, Nadiad-387 001, India

**Keywords:** Domperidone, floating matrix tablet, floating lag time, total floating time

## Abstract

Floating matrix tablets of domperidone were developed to prolong gastric residence time and thereby increased drug bioavailability. Domperidone was chosen as a model drug because it is poorly absorbed from the lower gastrointestinal tract. The tablets were prepared by wet granulation technique, using polymers such as hydroxypropylmethylcellulose K4M, carbopol 934P, and sodium alginate, either alone or in combination, and other standard excipients. Tablets were evaluated for physical characteristics viz. hardness, % friability, floating capacity, weight variation and content uniformity. Further, tablets were evaluated for *in vitro* release characteristics for 24 h. *In vitro* release mechanism was evaluated by linear regression analysis. Floating matrix tablets based on combination of three polymers namely; hydroxypropylmethylcellulose K4M, carbopol 934P and sodium alginate exhibited desired floating and prolonged drug release for 24 h. Carbopol loading showed negative effect on floating properties but were found helpful to control the release rate of drug.

Oral sustained release dosage forms (SRDFs) have been developed for the past three decades due to their considerable therapeutic advantages[1]. However, this approach has not been suitable for a variety of important drugs, characterized by a narrow absorption window in the upper part of the gastrointestinal tract (GIT), i.e. stomach and small intestine due to the relatively short transit time of the SRDFs in these anatomical segments. Thus, after only a short period (< 6 h), the SRDF lefts the upper GIT and the drug is released in nonabsorbing distal segments of the GIT. This results in a short absorption phase that is often accompanied by lesser bioavailability. It was suggested that compounding narrow absorption window drugs in a unique pharmaceutical dosage form with gastroretentive properties would enable an extended absorption phase of these drugs. After oral administration, such a dosage form would be retained in the stomach and release the drug there in a sustained manner, so that the drug could be supplied continuously to its absorption sites in the upper GIT. This mode of administration would best achieve the known pharmacokinetic and pharmacodynamic advantages of SRDFs for these drugs[2,3]. The need for gastroretentive dosage forms (GRDFs) has led to extensive efforts in both academia and industry towards the development of such drug delivery systems[4]. These efforts resulted in GRDFs that were designed in large part based on the approaches like: (a) low density form of the dosage form that causes buoyancy on the gastric fluid in the stomach[5]; (b) high density dosage form that is retained in the bottom of the stomach; (c) bioadhesion to the stomach mucosa[6]; (d) lowered motility o the GIT by concomitant administration of drugs or pharmaceutical excipients[7]; (e) expansion by swelling or unfolding to a large size which limits emptying of the dosage form through the pyloric sphincter[8].

Domperidone is a synthetic benzimidazole compound that acts as a dopamine D2 receptor antagonist. Its localization outside the blood-brain barrier and antiemetic properties has made it a useful adjunct in therapy for Parkinson's disease. There has been renewed interest in antidopaminergic prokinetic agents since the withdrawal of cisapride, a 5-HT4 agonist, from the market. Domperidone is also used as a prokinetic agent for treatment of upper gastrointestinal motility disorders[9,10]. It continues to be an attractive alternative to metoclopramide because it has fewer neurological side effects. Patients receiving domperidone or other prokinetic agents for diabetic gastropathy or gastroparesis should also be managing diet, lifestyle, and other medications to optimize gastric motility[11]. It is rapidly absorbed from the stomach and the upper part of the GIT by active transport[12], after oral administration, and few side effects have been reported[9,10]. It is a weak base with good solubility in acidic pH but in alkaline pH solubility is significantly reduced. Oral controlled release dosage forms containing drug, which is a weak base, are exposed to environments of increasing pH and poorly soluble freebase may get precipitated within the formulation in the intestinal fluid. Precipitated drug is no longer capable of being release from formulation[13,14]. The short biological half-life of the drug (7 h) also favors development of a sustained release formulation.

Based on this, an attempt was made through this investigation to formulate floating matrix tablets of domperidone using different polymers and their combinations. The prepared tablets were evaluated for physical characteristics such as hardness, thickness, % friability, floating capacity, weight variation and content uniformity. All the tablets were evaluated for *in vitro* release characteristics.

## MATERIALS AND METHODS

Domperidone was obtained as gift sample (Mann Pharmaceutical Ltd., Mehsana, India). Hydroxypropylmethylcellulose K4M (HPMC K4M), carbopol 934P were received as gift sample from Torrent Research Center (Gandhinagar, India). Sodium alginate (SA), sodium bicarbonate, lactose were obtained commercially from S. D. Fine Chemicals, (Mumbai, India) and used as received.

### Preparation of domperidone floating tablets:

Domperidone was mixed with required quantity of polymer (HPMC K4M or carbopol 934P or SA), sodium bicarbonate and lactose in mortar for 5 min by using a spatula. Isopropyl alcohol was added drop wise till suitable mass for granulation was obtained. The wet mass was granulated through sieve 40#. The granules were dried at room temperature (35°) for 1 h, and then blended with talc and magnesium stearate in the weight proportion as mentioned in Table 1 and compressed on 10-station rotary tablet compression machine (Rimek, Kadi, India) using a 8-mm standard flat-face die punch set.

**TABLE 1:** COMPOSITION OF PREPARED BATCHES

Batch Code	Dom	HPMC K4M	Carbopol 934P	SA	SB	Lac	PEG 4000	MS	Talc	Total Weight
MH1	30	36	-	-	20	88.6	-	1.8	3.6	180
MH2	30	54	-	-	20	70.6	-	1.8	3.6	180
MH3	30	72	-	-	20	52.6	-	1.8	3.6	180
MH4	30	90	-	-	20	34.6	-	1.8	3.6	180
MH5	30	108	-	-	20	16.6	-	1.8	3.6	180
MC1	30	-	18	-	20	106.6	-	1.8	3.6	180
MC2	30	-	36	-	20	88.6	-	1.8	3.6	180
MC3	30	-	45	-	20	79.6	-	1.8	3.6	180
MC4	30	-	54	-	20	70.6	-	1.8	3.6	180
MC5	30	-	72	-	20	52.6	-	1.8	3.6	180
MS1	30	-	-	18	20	106.6	-	1.8	3.6	180
MS2	30	-	-	36	20	88.6	-	1.8	3.6	180
MS3	30	-	-	45	20	79.6	-	1.8	3.6	180
MS4	30	-	-	54	20	70.6	-	1.8	3.6	180
MS5	30	-	-	72	20	52.6	-	1.8	3.6	180
T1	30	36	9	45	20	34.6	-	1.8	3.6	180
T2	30	36	18	45	20	25.6	-	1.8	3.6	180
T3	30	36	27	45	20	16.6	-	1.8	3.6	180
S1	30	36	9	45	20	25.6	9	1.8	3.6	180
S2	30	36	9	45	20	21.1	13.5	1.8	3.6	180
S3	30	36	9	45	20	16.6	18	1.8	3.6	180

*Quantities given for each tablet in mg; Dom: domperidone, SA: sodium alginate as gelling agent, SB: sodium bicarbonate as a gas forming agent, Lac: lactose as a diluent, PEG: Polyethylene glycol 4000 as a solubilizing agent and MS: magnesium stearate as a lubricant

### Physical characterization:

The fabricated tablets were characterized for weight variation (n=20), hardness (n=6, Monsanto hardness tester), thickness using a screw-gauge micrometer (Campbell Electronics, Mumbai, India) and % friability (n=20, Roche friabilator, Electrolab, Mumbai, India).

### Assay of tablets:

Twenty tablets from each batch were weighed and powdered. Powder equivalent to 30 mg of domperidone was accurately weighed and transferred into a 100 ml volumetric flask and dissolved in a suitable quantity of 0.1 N HCl. The prepared solution was diluted up to 100 ml with 0.1 N HCl and sonicated for 60 min. Five milliliters of the resulting solution was diluted to 100 ml with 0.1 N HCl to get a concentration in the range of 15 μg/ml. A portion of the sample was filtered through 0.45 μ membrane filter and analyzed by Shimadzu UV-1700 UV/Vis double-beam spectrophotometer (Kyoto, Japan) at 284 nm.

### Floating capacity:

The *in vitro* buoyancy was determined by floating lag times as per the method described by Rosa *et al*[15]*.* The tablets were placed in a 100 ml beaker containing 0.1 N HCl. The time required for the tablet to rise to the surface and float was determined as floating lag time. The experiments were conducted in triplicate. Total floating times were measured during *in vitro* dissolution studies.

### *In vitro* dissolution studies:

The release rate of domperidone from floating tablets (n=3) was determined as per British Pharmacopoeia (BP) using dissolution Testing Apparatus 2 (paddle method). The dissolution test was performed using 900 ml of 0.1N HCl, at 37±0.5° and 50 rpm. A sample (5 ml) of the solution was withdrawn from the dissolution apparatus hourly for 24 h, and the samples were replaced with fresh dissolution medium. The samples were filtered through 0.45 μ membrane filter and diluted to a suitable concentration with 0.1N HCl. Absorbance of these solutions was measured at 284 nm using a Shimadzu UV-1700 UV/Vis double-beam spectrophotometer (Kyoto, Japan). Duration of time the tablet constantly float on dissolution medium were noted as total floating time

## RESULTS AND DISCUSSION

Weight variation data of the prepared tablets indicated no significant difference in the weight of individual tablet from the average value. Hardness of the prepared tablets was observed to be within the range of 3.5±0.9 to 4.7±0.7 kg/cm^2^. Thickness of all the tablets was found in the range of 2.80±0.42 to 2.92±0.46 mm. Friability of all the tablets was found below 1%. The drug content in all the batches of domperidone floating tablets was in the range of 95 to 105% (i.e., a variation of ±5%). This ensured the uniformity of the drug content in the tablets (Table 2).

**TABLE 2:** EVALUATION OF PREPARED BATCHES

Batch Code	Hardness (Kg/cm^2^)	Friability (%)	Weight (mg)	Content (%)	Floating	

					Lag time (s)	Total time (h)
MH1	4.3 (0.4)	0.07	183 (2.5)	99.24	5	>12
MH2	4.8 (0.5)	0.04	185 (2.7)	98.64	11	>12
MH3	4.6 (0.6)	0.13	178 (1.4)	100.17	13	>12
MH4	4.7 (0.5)	0.04	181 (1.4)	99.12	18	>12
MH5	3.5 (0.3)	0.11	182 (2.9)	100.46	40	>12
MC1	4.5 (0.4)	0.08	177 (2.3)	99.39	5	4
MC2	4.6 (0.6)	0.06	179 (1.3)	98.53	90	4
MC3	4.1 (0.1)	0.13	180 (1.2)	100.31	120	6
MC4	4.1 (0.2)	0.04	177 (3.6)	99.52	138	3
MC5	4.3 (0.3)	0.11	182 (2.8)	100.16	140	2
MS1	4.3 (0.1)	0.08	178 (3.2)	99.34	5	2
MS2	4.5 (0.4)	0.07	184 (3.5)	99.13	13	5
MS3	4.3 (0.4)	0.11	183 (2.5)	101.01	18	>12
MS4	4.4 (0.2)	0.24	175 (1.3)	99.23	19	>12
MS5	4.3 (0.3)	0.13	182 (2.4)	100.43	22	>12
T1	4.4 (0.3)	0.08	181 (2.7)	99.46	5	5
T2	4.5 (0.6)	0.06	183 (2.9)	99.75	10	3
T3	4.3 (0.4)	0.13	184 (3.1)	101.01	13	3
S1	4.5 (0.3)	0.23	182 (2.6)	99.34	6	24
S2	4.3 (0.6)	0.07	180 (3.2)	99.31	8	20
S3	4.7 (0.3)	0.21	184 (2.4)	101.41	14	18

*The figures in parenthesis indicate standard deviation

Floating capacity of fabricated tablets was determined in 0.1N HCl, and the results are presented in Table 2. The tablets of all batches exhibited floating lag time less than 150 s. The tablets of carbopol 934P batches exhibited more floating lag time compared to other batches. Combination of three polymers showed no significant effect on floating lag time. Tablets formulated from carbopol 934P exhibited total floating time less then 7 h. This might be due to high affinity of carbopol toward water that promotes water penetration in tablet matrices leading to increased density. Partial replacement of carbopol 934P with polyethylene glycol 4000 increases total floating time because of reduces in density.

**Fig. 1:**
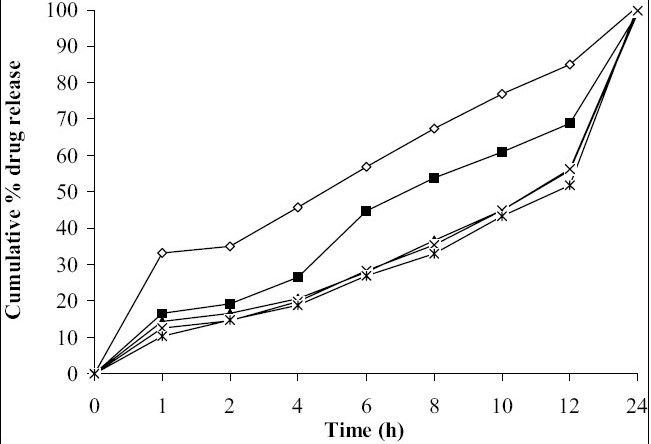
Effect of concentration of HPMC K4M on drug release profile Batch MH1 (-◇-), Batch MH2 (-■-), Batch MH3 (-▲-), Batch MH4 (-×-), Batch MH5 (-*-)

**Fig. 2:**
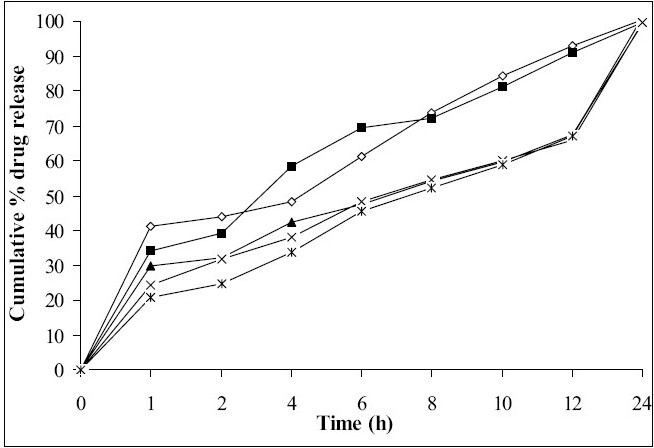
Effect of concentration of Carbopol 934 on drug release profile Batch MC1 (-◇-), Batch MC2 (-■-), Batch MC3 (-▲-), Batch MC4 (-×-), Batch MC5 (-*-)

**Fig. 3:**
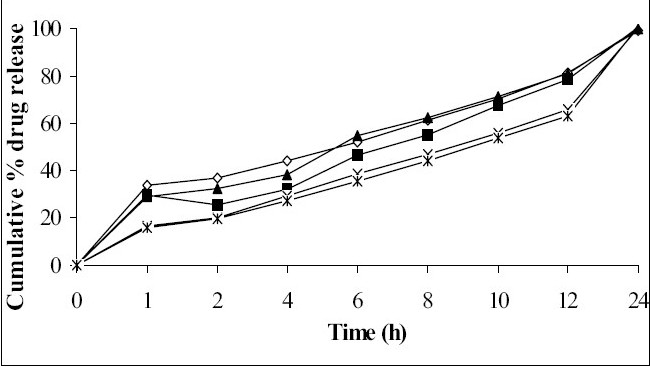
Effect of concentration of Sodium alginate on drug release profile. Batch MS1 (-◇-), Batch MS2 (-■-), Batch MS3 (-▲-), Batch MS4 (-×-), Batch MS5 (-*-)

*In vitro* dissolution studies showed that as the concentration of HPMC K4M was increased, drug release rate was decreased (fig. 1). Tablets of batch MH1 not showed good dissolution profile and about 40% of drug was released in 1 h, while tablets of batch MH2 released the drug in controlled manner at minimum level of HPMC content (30% w/w of tablet weight). As the concentration of carbopol 934P was increased drug release rate was decreased (fig. 2), this might be due to higher affinity of carbopol to water produce layer over tablet, which prevent dissolution of drug. Dissolution profiles of batch MS1 to MS3 were not good because high amount of drug release (30 to 36%) at 1 h. As the concentration of Sodium alginate was increased drug release rate was decreased (fig. 3).

**Fig. 4:**
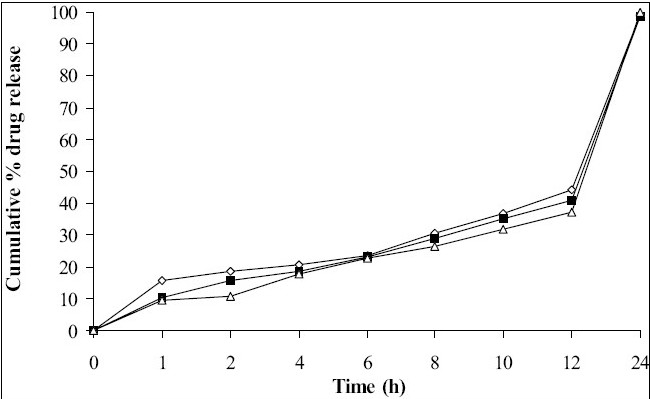
Effect of concentration of three polymers on drug release profile. Batch T1 (-◇-), Batch T2 (-■-), Batch T3 (-Δ-)

**Fig. 5:**
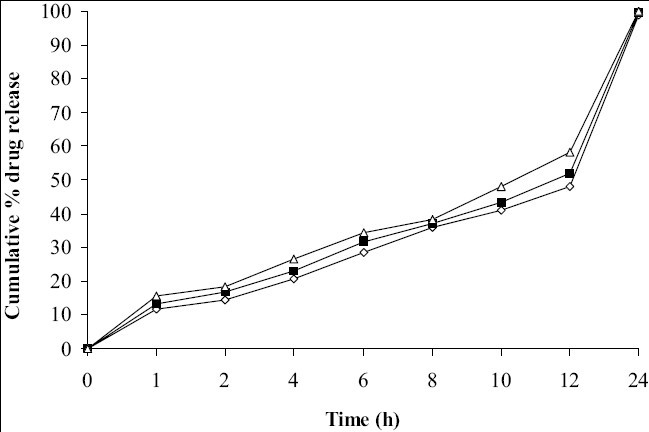
Effect of soubilizing agent on drug release profile. Batch S1 (-◇-), Batch S2 (-■-), Batch S3 (-Δ-)

Tablets prepared from combination of three polymers exhibited reduction of dissolution rate as the concentration of carbopol 934P increased (fig. 4). It might due to high affinity of water to carbopol compared to HPMC and SA. Hence, nine mg carbopol 934P per tablets was used for further study. As the concentration of PEG 4000 increased in tablet formulation dissolution rate was increased, it may be due to PEG 4000 create pores by solubizing itself, which was helpful for penetration of dissolution medium in matrix of tablets and helpful to increase buoyancy of tablets for 24 h. Concentration of PEG 4000 above 9 mg per tablets showed insignificant effect on dissolution rate may be due to localize effect of PEG 4000 (fig. 5). Fabricated tablets showed weight variation, hardness and uniformity of drug content within acceptable limits. A lesser floating lag time and desired total floating duration could be achieved by varying the amount of gas forming agent and using different polymer combinations.
